# Neural circuits and nicotinic acetylcholine receptors mediate the cholinergic regulation of midbrain dopaminergic neurons and nicotine dependence

**DOI:** 10.1038/s41401-019-0299-4

**Published:** 2019-09-25

**Authors:** Cheng Xiao, Chun-yi Zhou, Jin-hong Jiang, Cui Yin

**Affiliations:** 1 School of Anesthesiology, Xuzhou Medical University, Xuzhou, 221004 China; 2 Jiangsu Province Key Laboratory in Anesthesiology, Xuzhou Medical University, Xuzhou, 221004 China

**Keywords:** midbrain DA neurons, mesopontine, cholinergic neurons, nicotinic acetylcholine receptors, neural circuits, nicotine reward and dependence, smoking intervention

## Abstract

Midbrain dopaminergic (DA) neurons are governed by an endogenous cholinergic system, originated in the mesopontine nuclei. Nicotine hijacks nicotinic acetylcholine receptors (nAChRs) and interferes with physiological function of the cholinergic system. In this review, we describe the anatomical organization of the cholinergic system and the key nAChR subtypes mediating cholinergic regulation of DA transmission and nicotine reward and dependence, in an effort to identify potential targets for smoking intervention. Cholinergic modulation of midbrain DA systems relies on topographic organization of mesopontine cholinergic projections, and activation of nAChRs in midbrain DA neurons. Previous studies have revealed that α4, α6, and β2 subunit-containing nAChRs expressed in midbrain DA neurons and their terminals in the striatum regulate firings of midbrain DA neurons and activity-dependent dopamine release in the striatum. These nAChRs undergo modification upon chronic nicotine exposure. Clinical investigation has demonstrated that partial agonists of these receptors elevate the success rate of smoking cessation relative to placebo. However, further investigations are required to refine the drug targets to mitigate unpleasant side-effects.

## Introduction

Cigarette smoking causes the most preventable diseases worldwide [1]. Nicotine is a bioactive compound in cigarettes that exerts rewarding effects by activating nicotinic acetylcholine receptors (nAChRs) in the central nervous system. Repetitive nicotine intake modifies plasticity in the central nervous system, leading to nicotine dependence [2]. Among the brain regions responsive to nicotine, the midbrain contains dopaminergic (DA) neurons, which have been implicated in a wide range of physiological functions, including reward processing, reinforcement learning, aversion avoidance, and motivation [3, 4]. Therefore, the midbrain is unique in that it is the target of nicotine for the development and maintenance of nicotine dependence.

Midbrain neurons are governed by the endogenous cholinergic system, originating in the mesopontine nuclei [5–8]. Nicotine hijacks nAChRs and interferes with the physiological function of endogenous ACh, and thus identifying and characterizing the key ACh receptors that mediate the cholinergic regulation of DA transmission may advance our understanding of the circuit mechanisms underlying nicotine dependence.

In this article, we review the topographic organization of the cholinergic system that governs midbrain DA neurons, the composition of ACh receptors that mediate the cholinergic modulation of midbrain neurons, the subtypes of nAChRs modified by chronic exposure to nicotine, and the subtypes of nAChRs implicated in nicotine dependence.

## Topographic organization of the cholinergic system for the regulation of midbrain DA neurons

Midbrain DA neurons are distributed in the ventral tegmental area (VTA) and the substantia nigra pars compacta (SNc), and they receive dense cholinergic innervation from mesopontine cholinergic nuclei, including the pedunculopontine tegmental nucleus (PPN) and the laterodorsal tegmental nucleus (LDT) [5–8]. Previous studies have revealed that the mesopontine cholinergic innervation of midbrain DA neurons is topographically organized and forms anatomical substrates for the independent regulation of different behaviors by the mesopontine cholinergic system [6, 8, 9].

### Anatomy of the PPN and LDT

**Fig. 1:**
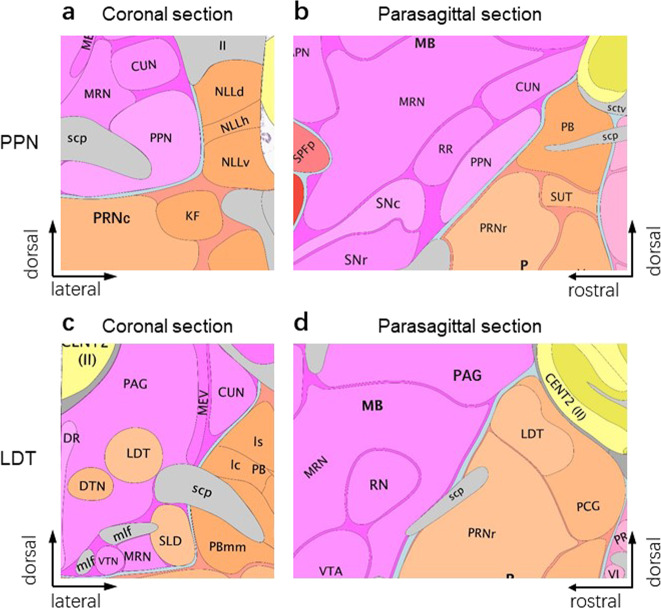
Anatomy of the pedunculopontine nucleus (PPN) and the lateral dorsal tegmental nucleus (LDT). **a** Coronal section containing the pedunculopontine nucleus (PPN). **b** Parasagittal section containing the PPN. **c** Coronal section containing the LDT. **d** Parasagittal section containing the LDT. CUN: cuneiform nucleus. DR: dorsal raphe. DTN: dorsal tegmental nucleus. mlf: medial longitudinal fascicle. MRN: midbrain reticular nucleus. PAG: periaqueductal gray. PB: parabrachial nucleus (lc: lateral part, central lateral; ls: lateral part, superior lateral). PCG: pontine central gray. PRNc: pontine reticular nucleus, caudal. PRNr: pontine reticular nucleus, rostral. RN: raphe nucleus. RR: retrorubral area. Scp: superior cerebellar peduncle. SLD: sublaterodorsal nucleus. SNc: substantia nigra pars compacta. SNr: substantia nigra pars reticulata. VTA: ventral tegmental area. Image credit: Allen Adult Mouse Brain Reference Atlas

The PPN is located in the pons of the upper brainstem, and its border can be demarcated by staining with an antibody against choline acetyltransferase, a marker protein of cholinergic neurons [6]. Its rostral-ventral end begins just below the red nucleus and posterior to the SN, and its dorsal-posterior edge is in front of the anterior parabrachial nucleus. The PPN is located medial to the medial lemniscus and the superior cerebellar peduncle, lateral to the brachium conjunctivum, ventral to the retrorubral area of the midbrain reticular nucleus and the cuneiform nucleus, and dorsal to the pontine reticular nucleus and the parabrachial nucleus (Fig. 1a, b, atlas.brain-map.org). Neurons in the PPN are heterologous in density, size, and neurochemistry. The PPN is divided into the rostral half, the pars dissipata (PPNd), and the caudal half, the pars compacta (PPNc). The PPNd and PPNc mainly consist of small GABAergic neurons and large cholinergic and glutamatergic neurons, respectively [10]. It is noteworthy that the cholinergic neurons also contain nitric oxide synthase, substance P and atrial natriuretic peptide [10]. Similar to the cuneiform nucleus, the PPN is a major component of the mesencephalic locomotor region [11, 12]. The electrical stimulation of this region promotes locomotion [11]. A recent elegant study utilizing a cell-specific optogenetic technique revealed that PPN glutamatergic and GABAergic neurons respecitvely facilitate and inhibit movement in mice [12].

The LDT is medial-posterior to the PPN. It is embedded in the pontine central gray and lies between the caudal part of the dorsal raphe and the parabrachial nucleus and ventral to the caudal part of the ventral periaqueductal gray (Fig. 1c, d, atlas.brain-map.org). Similar to the PPN, the LDT contains cholinergic, glutamatergic and GABAergic neurons [6, 7].

### Cholinergic modulation of the midbrain

PPN and LDT cholinergic neurons release acetylcholine (ACh) into the midbrain and regulate neuronal activity via activating nicotinic and muscarinic ACh receptors (nAChRs and mAChRs) in these neurons. nAChRs are ligand-gated cation channels, and each individual receptor is composed of five subunits. Each subunit has four transmembrane domains, of which the second transmembrane domain faces the pore of the channel. nAChR subunits in the central nervous system include α2-7 and β2-4 [13, 14]. These subunits form either homomeric pentamers (i.e., α7) or heteromeric pentamers (which include two or three α-subunits and three or two β-subunits). mAChRs belong to a family of seven-transmembrane G-protein coupled receptors that include five members (M1–M5) [15]. M1, M3, and M5 receptors are coupled with Gq. M2 and M4 receptors are coupled with Gi and inhibit adenosine monophosphate cyclase, potassium channels, and calcium channels, etc.

### PPN and LDT cholinergic neurons regulate SNc and VTA neurons by different patterns

The cholinergic neurons in the PPN and LDT regulate both SNc and VTA neurons through activating acetylcholine receptors in these neurons [8]. Combining optogenetic and brain slice patch-clamp techniques, Xiao et al. [8] demonstrated that 5–10 s of optogenetic stimulation of cholinergic projections from the PPN and LDT evokes inward currents and increases the firing rates in both SNc and VTA neurons. The effects can be blocked by a nAChR antagonist but not by antagonists of mAChRs and GABA_A_ receptors. Interestingly, in some midbrain neurons, blocking AMPA and NMDA receptors significantly attenuates cholinergic responses. These data suggest that the cholinergic responses are mediated by nAChRs in the midbrain neurons and in the glutamatergic terminals that synapse onto these midbrain neurons. This finding is consistent with that of a previous electrophysiological study in which the authors applied electrical stimulation to the PPN and found that blocking nAChRs attenuated glutamatergic responses in the VTA [16] and the SNc [17]. Using in vivo single-unit recordings from anesthetized rats, Dautan et al. [5] applied optogenetic stimulation to cholinergic neurons in the PPN and the LDT and observed the excitation of VTA DA neurons, which are regulated by AChRs (the effect is blocked by locally applied atropine and mecamylamine). Although some PPN cholinergic neurons are glutamatergic or GABAergic neurons [18] and corelease ACh with glutamate or GABA, using optogenetic techniques to stimulate PPN cholinergic terminals in the midbrain does not evoke the release of glutamate or GABA from the terminals [5, 8], indicating that PPN cholinergic neurons that contain ACh and glutamate or GABA do not project to the midbrain. Viral vector-assisted retrograde neuronal tracing shows that cholinergic neurons account for most PPN neurons that project to the ventral SNc [8] but only a minority of LDT neurons that project to the VTA [8]. Instead, most VTA-projecting LDT neurons are glutamatergic [19]. Therefore, PPN and LDT cholinergic neurons may selectively innervate certain regions in the midbrain.

### Topographic organization of PPN and LDT cholinergic projections to the SNc and VTA

**Fig. 2:**
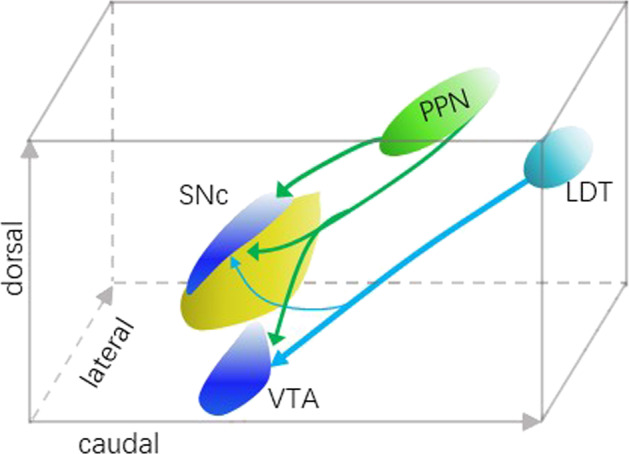
Topographic organization of the midbrain cholinergic system. The rostral and caudal parts of the PPN project to the lateral SNc and medial SNc and lateral VTA, respectively. The LDT projects preferentially to the VTA rather than the medial SNc. Blue represents DA neurons, while cyan and green represent cholinergic neurons in the LDT and PPN, respectively. In each individual nucleus, the color gradually becomes lighter from the medial to the lateral portion

PPN and LDT cholinergic neuron projections to the SNc and VTA display particular patterns of topographic organization [6]. In the lateral-to-medial dimension, midbrain DA neurons are distributed in the lateral SNc, medial SNc and VTA, which respectively receive cholinergic afferents from the PPNd, PPNc, and LDT [6]. In general, PPNd neurons mainly project to the lateral SNc; PPNc neurons project to both the SNc and VTA, while LDT neurons mainly project to the VTA (Fig. 2). The distribution of cholinergic neurons that project to the substantia nigra (SN) shows a gradient along the anterior-posterior axis of the PPN. SN-projecting cholinergic neurons account for ~35%, 25%, and 15% of neurons in the rostral, middle, and caudal thirds of the PPN [20], respectively, but are rarely found in the LDT [21]. VTA-projecting neurons are distributed throughout the PPN and LDT with higher densities in the PPNc and LDT, in which cholinergic neurons are densely distributed [21]. This topographic feature of mesopontine cholinergic afferents to the midbrain is the anatomical basis for the differential regulation of locomotion and reward behaviors. PPNc cholinergic neurons that project to the SNc and VTA regulate locomotion and reward [8], respectively. LDT cholinergic neurons regulate both SNc and VTA neurons but regulate behaviors in a pattern different from that by which PPN cholinergic neurons do [8]: stimulating LDT cholinergic terminals in the VTA elicits reward-related behaviors, while stimulating those in the SNc does not promote locomotion.

### PPN cholinergic projections differentially regulate the lateral and medial SNc

The dorsal SNc is divided into medial and lateral portions by the oculomotor nerve. DA neurons in these two portions are distinct in electrophysiological characteristics, such as membrane potential, the size of hyperpolarization-activated cyclic nucleotide-gated cation channels, and spontaneous firing rate [9]. This study revealed that PPN cholinergic neurons form disparate types of connections with medial and lateral SNc DA neurons. In the lateral SNc, PPN cholinergic terminals release ACh and then activate nAChRs on DA neurons and glutamatergic terminals, resulting in excitation of DA neurons, similar to another study [8]. In the medial SNc, PPN cholinergic terminals release ACh (activating nAChRs on DA neurons and GABAergic terminals) or corelease GABA (activating nAChRs and GABA_A_ receptors on DA neurons), leading to inhibition of DA neurons. At the behavioral level, the stimulation of PPN cholinergic terminals in the lateral and medial SNc enhances and inhibits locomotion, respectively. This is the first study to unambiguously demonstrate that some mesopontine cholinergic neurons corelease ACh and GABA and are critically implicated in the regulation of downstream neurons and related behaviors.

Estakhr et al. [9] demonstrated the corelease of ACh and GABA from PPN cholinergic terminals in the SNc of ChAT-Cre mice, which is different from what was shown by another study using ChAT-Cre rats [8]. In that study, AAV5 was used to limit the transfection area, and only 50%–60% of cholinergic neurons in the PPNc were successfully transduced with channelrhodopsin [8]. It has been shown that the PPNc contains fewer GABAergic neurons than the PPNd [10]. Therefore, the different results between these two similar optogenetic studies may have resulted from anatomical variation between species or differences in the transduction efficiency of viral vectors or the locations of the optogenetically labeled neurons.

Note that the properties of PPN-LDT cholinergic projections to the midbrain differ from those of medial habenular (MHb) cholinergic projections to the interpedunculopontine nucleus (IPN). Habenular cholinergic neurons corelease ACh and glutamate and stimulate IPN neurons by activating both glutamate receptors and nAChRs [22]. This supports previous histological studies showing that a large proportion of MHb neurons produce ACh and glutamate [23].

## Acetylcholine receptor subtypes mediate the cholinergic modulation of the midbrain

The stimulation of cholinergic afferents results in multiphasic alterations in neuronal firing in vivo [24–27]. Stimulating PPN neurons with kainate increases the firing rate of DA neurons in the ipsilateral substantia nigra by activating nAChRs [27]. Foster and Blaha applied 35 Hz electrical stimulation to the LDT and PPN every other second for 1 min and recorded triphasic alterations (the first increase, second decrease, and third sustained elevation) in dopamine levels in the nucleus accumbens (NAcc) and the caudate putamen (CPu), indicating that stimulating these cholinergic nuclei modulates midbrain DA neurons [25, 26]. Pharmacological evidence has revealed that these responses are, respectively, mediated by nAChRs in the VTA/SN, mAChRs in the LDT/PPN, and mAChRs in the VTA/SN. Therefore, nAChRs and mAChRs in the VTA/SN mediate the fast and slow excitation of VTA/SN neurons, respectively, following stimulation of the PPN and LDT. It is noteworthy that PPN and LDT cholinergic projections not only regulate midbrain DA neurons but are also modulated by nicotine. In the PPN and LDT, nicotine activates nAChRs in non-cholinergic neurons and indirectly modulates cholinergic neurons [28]. This circuitry contributes to nicotine reinforcement learning because lesions of PPN cholinergic neurons or the inhibition of nAChRs in the PPN reduces nicotine self-administration in rats [29].

### Subtypes of nAChRs in midbrain neurons

Accumulating evidence has demonstrated that M5-type mAChRs in midbrain DA neurons mediate the sustained increase of dopamine release in the striatum following the electrical stimulation of the PPN [30–32]. In midbrain DA neurons, the subtypes of nAChRs are more complicated than those of mAChRs. Smoking-relevant concentrations of nicotine activate nAChRs, increase the firing rate of DA neurons in the VTA [33–35] and SNc [36–38], and evoke prolonged irregular firing in these neurons [38]. The excitation of VTA DA neurons is much stronger than that of SNc DA neurons [36, 38], suggesting that nAChR subtypes in VTA and SNc DA neurons and the neural circuitry in the VTA and SNc may be different.

#### Expression of nAChR subtypes in midbrain neurons

Using double-labeling in situ hybridization, Azam et al. [39] identified nAChR subunit messenger RNAs (mRNAs) expressed in SN and VTA DA neurons. They found that these DA neurons contain α2-7 and β2-4 subunits. Specifically, almost all SNc and VTA DA neurons contain α2, α4, α5, α6, β2, and β3 nAChR mRNAs; some also contain α3 and α7 mRNAs and few neurons contain β4 mRNA. In the SN, α4, β2, α7, and β4 mRNAs are also detected in non-DA neurons. The results are consistent with those of another study showing that 6-OHDA-induced lesions in SNc DA neurons eliminate α3, α5, α6, and β4 subunit mRNAs but only reduce the levels of α4, α7, β2, and β3 subunit mRNAs in the SN. Thus, α3, α5, α6, and β4 subunits are selectively expressed in SNc DA neurons, while α4, α7, β2, and β3 subunits consist of major nAChR subtypes in both DA and non-DA neurons [40]. The selective expression of α3, α5, α6, and β3 subunits in midbrain DA neurons has also been supported by many other studies [41–45].

#### Functional nAChRs in midbrain DA neurons

To understand how nAChR subunits integrate and form functional receptors to regulate midbrain DA neurons, many studies have utilized ex vivo patch-clamp recordings to define ACh responses in midbrain DA neurons with drugs selective for nAChR subtypes and with mouse lines having genetically modified nAChR subunits. These studies have delineated four types of ACh responses in midbrain DA neurons [46–49]. The first type is sensitive to dihydro-β-erythroidine, an antagonist of nAChRs containing α4β2 subunits. The second type is mediated by α7-containing nAChRs and blocked by methyllycaconitine or α-bungarotoxin. The third type is the combination of the first and second types. The fourth type is sensitive to low concentrations of mecamylamine, which preferentially blocks α3β4-containing nAChRs.

α4β2- and α7-containing nAChRs may differ between VTA and SNc DA neurons. Electrophysiological analysis of nAChR currents, autoradiography of [^125^I]-α-bungarotoxin binding (specific for α7 nAChRs), and in situ hybridization have revealed that the major components for ACh-evoked responses in midbrain DA neurons are mediated by α4β2 nAChRs, and VTA DA neurons contain higher levels of α7 nAChRs than SNc DA neurons [48, 50]. The activation of α4β2 nAChRs with partial and full agonists promotes the firing rate [51, 52] and facilitates burst firing [52] in VTA DA neurons.

Klink et al. [47] coupled single-cell PCR with electrophysiological recordings and further divided α4β2-containing nAChRs into two subtypes: α4α5α6β2-containing nAChRs (sensitive to both dihydro-β-erythroidine and α-conotoxin MII) and α4α5β2-containing nAChRs (sensitive to dihydro-β-erythroidine, but not to α-conotoxin MII). The presence of the α5 subunit facilitates the assembly of α4 nAChRs, increases the levels of α4 subunit-containing nAChRs by 60%, and slows down the desensitization of these nAChRs in the VTA [53]. Conversely, a loss-of-function mutation in the α5 subunit or the knock-out of the α5 subunit dramatically reduces the nicotine sensitivity of nAChRs, and in these situations, higher levels of nicotine intake are required to induce rewarding effects [54–59].

A bacterial artificial chromosome (BAC)-based genetic modification strategy was employed to introduce gain-of-function α6 subunits (L9′S) into mice [43]. In this transgenic mouse line, neurons expressing α6-containing nAChRs are selectively stimulated by low concentrations of nicotine that are unable to excite midbrain DA neurons in wild-type mice. Using this mouse line, Drenan et al. [43] provided functional evidence showing that midbrain DA neurons, but not GABAergic neurons, possess α6-containing nAChRs. Another study demonstrated that either blocking α6-containing nAChRs with α-conotoxin MII [H9A;L15A] or knocking out the α4 subunit reduces the prolonged excitation of VTA DA neurons induced by smoking-relevant concentrations of nicotine (100–500 nM) [60]. Thus, the α4 and α6 subunits are necessary for the nicotine-induced excitation of VTA DA neurons.

**Fig. 3:**
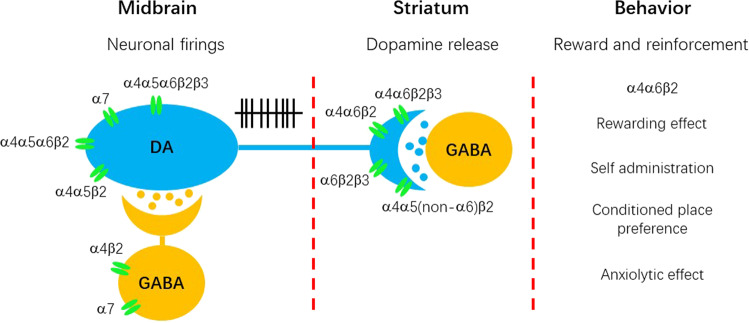
nAChR subtypes in midbrain neurons and their striatal terminals regulate rodent behaviors. nAChRs in midbrain DA (cyan) and GABAergic (yellow) neurons integrate to modulate the activity of DA neurons. nAChRs in striatal DA terminals determine the activity-dependent gating of dopamine release onto medium spiny neurons (yellow). Nicotine activates nAChRs in midbrain neurons and striatal DA terminals to cause nicotine reward and reinforcement

Therefore, the major nAChR subtypes that mediate the nicotinic excitation of midbrain DA neurons contain α4α5α6β2 and α4α5β2 subunits (Fig. 3).

#### Stoichiometry of nAChRs in midbrain DA neurons

In addition to subunit composition, the stoichiometry of nAChRs (i.e., the number of α- and β-subunits, specifically, α(3)β(2) or α(2)β(3)) also affects receptor function. In in vitro heterologous expression systems, the ratio of α4 to β2 nAChR subunits being transfected was adjusted from 1:10 to 10:1 by researchers to shift the stoichiometry from almost pure α4(2)β2(3) to almost pure α4(3)β2(2) [61–63]. These studies revealed that, compared with α4(3)β2(2) nAChRs, α4(2)β2(3) nAChRs have higher sensitivity to nicotinic agonists (nicotine, ACh and TC-2559), lower sensitivity to epibatidine and cytisine, smaller nicotinic and ACh currents, and more evident desensitization upon nicotine exposure. Thus, high-sensitivity nAChRs are those responsive to submicromolar concentrations of nicotine obtained from cigarette smoking.

### Subtypes of nAChRs in mesostriatal DA terminals

Like nAChRs in midbrain DA neuron somata, those in striatal DA terminals contain α6 subunits [43, 64]. In the striatum (including the NAcc and the CPu), both α4α6β2β3 and α4α6β2 nAChRs are detected [50, 64]. In β3 subunit knock-out mice, there are 76% fewer α6 subunits in the striatum, and respectively 34% and 42% fewer α3 and α6 subunits in the midbrain, than in wild-type mice, indicating that β3 subunits increase the incorporation of α6 subunits into α4β2-containing nAChRs [64]. The α-conotoxin analog E11A (α-CtxMII-E11A) binds primarily to α6α4β2β3 and α6β2β3 nAChRs in striatal DA terminals with a femtomolar and a picomolar affinity, respectively [65]. Compared with VTA DA neurons, SNc DA neurons are more susceptible to lesions by neurotoxins such as MPTP and 6-OHDA [66]. The fact that α6α4β2β3 nAChRs are more vulnerable to MPTP-induced lesions than α6β2β3 nAChRs [65] supports the notion that α6α4β2β3 nAChRs, but not α6β2β3 nAChRs, are among the major nAChRs in DA terminals originating from the SNc.

The magnitude of electrical stimulation-induced dopamine release exhibits regional differences: it is larger in the ventral striatum (the NAcc) than in the dorsal striatum (the CPu) [67–69]. Nicotine modulates dopamine release through two opposite mechanisms. After a short period of exposure, nicotine activates presynaptic nAChRs on DA terminals and enhances dopamine release evoked by low-frequency stimulation (mimicking tonic activity of DA neurons) in the striatum, whereas after longer exposure, nicotine desensitizes nAChRs and reduces dopamine release evoked by single stimulation, but amplifies dopamine release evoked by high-frequency (mimicking burst/phasic firing) electrical stimulation [69, 70]. This contrast suggests that the desensitization of nAChRs in DA terminals may play important roles in nicotine reinforcement.

It has been demonstrated that α4α6β2 nAChRs and α4(non-α6)β2 nAChRs are major receptors that, respectively, mediate the nicotinic regulation of dopamine release into the NAcc and the CPu [67, 71]. However, an assay of α-conotoxin analog E11A (α-CtxMII-E11A) binding [65] showed that there are α6α4β2β3 nAChRs in DA terminals in the CPu. Further investigations are warranted to address why α6α4β2β3 nAChRs significantly regulate dopamine release in the NAcc, but not in the CPu.

## Chronic nicotine upregulates nAChR subunits in both number and function

Acute exposure to nicotine activates nAChRs, while chronic nicotine exposure modifies nAChRs, conferring various physiological outcomes. Chronic nicotine exposure regimens include continuous nicotine administration [72–74], repeated intermittent exposure [75], self-administration [76], and yoked-nicotine administration [74]. To evaluate the effects of chronic nicotine on nAChRs, the levels of nAChRs were quantified with an epibatidine/nicotine binding assay [74, 75, 77] and by studying the levels of fluorescent protein-tagged nAChR subunits [72, 76]. Under different exposure paradigms, chronic nicotine similarly upregulates nAChRs, but with selectivity for nAChR subtypes, the stoichiometry of nAChRs, cell types, and cell compartments [72, 73, 78, 79].

### Chronic nicotine increases the levels of nAChR subunits

To enable the visualization and quantification of nAChRs containing α4 subunits in mice and to examine the dynamic alteration of these receptors during nicotine addiction and in different developmental stages, Nashmi et al. [72, 78] developed knock-in mice in which the α4 nAChR subunit is replaced with normally functioning and fluorescently tagged subunits (α4-eYFP). They found that chronic nicotine did not change α4-containing nAChRs in midbrain DA neurons but upregulated α4-containing nAChRs in midbrain GABAergic neurons and in the dorsal striatum [72]. Using the same mouse line, Renda et al. [76] exposed mice of different ages to a two-bottle choice oral nicotine self-administration paradigm for 5 days. They found that the amount of nicotine self-administered was significantly higher in 44-day-old and 54–60-day-old mice than in 66–86-day-old mice and that it showed a strong-positive correlation with the levels of α4-eYFP [76].

In comparison with α4β2 nAChRs, α6β2, α3β2, and α7 nAChRs are less sensitive in terms of being upregulated by chronic nicotine [77, 80, 81]. Although α5 subunits facilitate the assembly of α4β2 nAChRs, they attenuate the chronic nicotine-induced upregulation of α4β2 nAChRs [82]. In striatal DA terminals, α6β2-containing nAChRs are downregulated by chronic exposure to nicotine at a concentration threefold lower than that required to upregulate α4β2-containing nAChRs [83].

### Chronic nicotine also alters the stoichiometry of nAChRs

Several studies have revealed that chronic nicotine treatment enhances cationic currents evoked by 1 μM nicotine or 3–30 μM ACh in SNr GABAergic neurons but not in SNc DA neurons [72, 73]. This finding suggests that chronic nicotine can bias the stoichiometry of nAChRs to high-sensitivity (α(2)β(3)) ones. This notion is also consistent with previous in vitro studies showing that nicotine more efficiently upregulates α4(2)β2(3) nAChRs than α4(3)β2(2) nAChRs [61–63]. The effects of chronic nicotine treatment on the biophysical properties of nAChRs are controversial. Buisson and Bertrand [61] showed that α4(2)β2(3) nAChRs exhibit larger single-channel conductance and slower desensitization kinetics, but Lopez-Hernandez et al. [62] showed that α4(2)β2(3) nAChRs mediate smaller currents and display more evident desensitization upon acute nicotine exposure.

Gain-of-function α4 (L9′S) subunits were transduced into mouse VTA GABAergic neurons to directly enhance α4-containing nAChR function in these neurons, mimicking the chronic nicotine effect (upregulating nAChRs in number and sensitivity) [84]. This strategy increased the sensitivity of nAChRs to nicotine in GABAergic neurons, and conditioned place preference was induced by low concentrations of nicotine in these mice [84]. The results suggest that the cell-type selective upregulation of α4-containing nAChRs by chronic nicotine can promote nicotine-seeking behaviors.

## nAChR subtypes are involved in nicotine dependence

Nicotine dependence is a chronically relapsing behavioral disorder with typical manifestations of drug addiction, such as compulsive cravings for nicotine, a loss of control to limit nicotine intake, and withdrawal-like symptoms after access to nicotine is prevented [2, 85]. The rewarding and reinforcing effects of nicotine involve midbrain neurons and can be measured with nicotine-conditioned place preference and nicotine self-administration [2, 85]. Withdrawal symptoms after nicotine abstinence are regulated by the extended amygdala and MHb-IPN pathway [85, 86]. Accumulating evidence has shown that different subtypes of nAChRs play distinct roles in the effects of nicotine.

### α4 nAChRs

A nicotine-conditioned place preference (CPP) paradigm was used in two genetic mouse lines, one lacking the α4 subunit (α4 knock-out) and one carrying a gain-of-function mutation in the α4 subunit (α4 L9′A) [87]. The knock-out of α4 nAChRs eliminated responses to smoking-relevant concentrations of nicotine in VTA neurons, while α4 L9′A-containing nAChRs enabled the excitation of VTA neurons by nicotine at concentrations much lower than those achieved by cigarette smoking [87]. Consistently, the administration of nicotine at the concentration that establishes CPP in wild-type mice did not establish CPP in α4 knock-out mice, but nicotine induced CPP in α4 (L9′A) mice at a much lower concentration [87]. As the nAChRs of these mice are modified throughout the entire brain, the results may be inadequate to conclude that the contributing α4 nAChRs are in midbrain DA neurons. Conditionally deleting α4 nAChRs in the ventral midbrain using a viral vector-assisted Cre/loxP approach, Peng et al. [88] revealed that mice lacking α4 nAChRs in the ventral midbrain consumed more nicotine but did not exhibit nicotine CPP. These results seem contradictory, but the authors argue that the lack of α4 nAChRs may attenuate nicotine-induced aversive effects but increase the amount of nicotine required to stimulate the reward system. McGranahan et al. [89] restricted the genetic deletion of α4 subunits to DA neurons in mice without perturbing α4 nAChRs in GABAergic neurons. This manipulation eliminated nicotine CPP. The results further demonstrated that α4 nAChRs in DA neurons are necessary for the development of nicotine-seeking behavior.

### β2 nAChRs

α4 and β2 subunits form the major nAChR subtype in the midbrain and mediate the reinforcing effects of nicotine [46–49]. The genetic modification of the β2 subunit has similar effects as that of the α4 subunit. It has been demonstrated that β2 nAChRs are necessary to mediate nicotine responses in midbrain neurons, and knocking out these nAChRs dramatically reduces nicotine self-administration [90]. The re-expression of the β2 subunit in VTA neurons of β2 subunit knock-out mice restores nicotine-induced responses, including the excitation of VTA DA neurons, an increase in dopamine release in the NAcc, and nicotine self-administration [91, 92].

Several mechanisms may underlie the nicotine-induced stimulation of VTA DA neurons [35, 87, 90, 92–94]. First, nicotine directly activates nAChRs on DA neurons. Second, nicotine desensitizes nAChRs on local GABAergic neurons, leading to the disinhibition of DA neurons. Third, nicotine activates nAChRs in glutamatergic terminals that synapse onto DA neurons to cause the sustained excitation of DA neurons. An elegant study employed a cell-specific viral-vector strategy to re-express the β2 subunit in either VTA DA neurons, GABAergic neurons or both in β2 subunit knock-out mice and revealed that, in this mouse line, nicotine respectively excites, inhibits and enhances burst firing in VTA DA neurons in vivo and correspondingly causes transient rewarding, aversive, and reinforcing effects [95]. Their data suggest that the induction of burst firing in VTA DA neurons might be a prerequisite for the establishment of nicotine self-administration and requires the activation of β2 nAChRs on both DA and GABAergic neurons.

### α4 and α6 nAChRs

In the VTA, the α6 subunit is selectively expressed in DA neurons [41–45]. Exley et al. [96] demonstrated that the α4 and α6 subunits play different roles in the reinforcing effect of nicotine. Their data showed that nAChR α4 subunits are required to establish intracranial nicotine self-administration and to induce burst firing in VTA DA neurons, whereas both the α4 and α6 subunits are involved in the regulation of activity-dependent dopamine release in the NAcc. Therefore, α4 subunit-containing nAChRs in VTA DA neuron somata and α4 and α6 subunit-containing nAChRs in DA terminals in the NAcc control the activity of DA neurons and the release of dopamine, respectively. These two processes may, respectively, underlie nicotine self-administration and its long-term maintenance [96].

### α5-α3-β4 nAChRs

The genes that encode the α5, α3, and β4 nAChR subunits form a cluster in chromosome region 15q25, and some allelic variations in this gene cluster are risk factors for nicotine dependence [97, 98]. A single-nucleotide polymorphism (SNP) in the α5 subunit gene (CHRNA5) (rs16969968) increases the incidence of tobacco dependence, heavy smoking, and the early onset of smoking behaviors [97–100]. SNPs that have similar effects on nicotine dependence include rs6495308, rs578776, and rs1051730 in the α3 subunit gene (CHRNA3) [97, 101, 102] and rs1948 in the β4 subunit gene (CHRNB4) [103]. Consistent with these epidemiological studies, the knock-out of the α5 subunit increases nicotine self-administration in mice [86]. Although α5, α3, and β4 nAChR subunits are expressed in some midbrain DA neurons, the levels are much lower than those in the MHb and IPN [104]. Furthermore, the re-expression of the α5 subunit in the MHb-IPN pathway in α5 knock-out mice reverses the enhancement of nicotine self-administration [86]. Therefore, α5 nAChRs in the MHb-IPN pathway play critical roles in nicotine intake.

Both systemic and intra-IPN administration of mecamylamine, an antagonist of nAChRs, precipitates withdrawal symptoms in nicotine-dependent animals, but not in α5 and α2 knock-out mice [105], supporting the notion that α5 nAChRs in the MHb-IPN pathway regulate the expression of withdrawal symptoms in nicotine-dependent animals.

                                                                                                                                                                                                                                                             In conclusion, the cholinergic modulation of midbrain DA neurons relies on the topographic organization of PPN and LDT cholinergic projections (Fig. 2) and the activation of cholinergic receptors in midbrain DA neurons and their terminals in the NAcc and CPu (Fig. 3 and Table 1). nAChRs in midbrain DA neurons and GABAergic neurons are involved in the regulation of firing rates and patterns of DA neurons. In this process, the contributing nAChRs in DA neurons may contain α4, β2, α6, and α7 subunits, while those in GABAergic neurons express α4, β2, and α7 subunits. It is noteworthy that α4β2 nAChRs in midbrain GABAergic neurons have been revealed to play critical roles in the nicotine-induced sustained enhancement of firing rates and bursting firing patterns. The latter is essential for the development of nicotine self-administration. On the other hand, nAChRs containing α4, β2, α6 subunits on striatal DA terminals provide a gating mechanism for the adjustment of activity-dependent dopamine release, which is important for maintaining long-term nicotine intake. Therefore, the α4, β2, α6 subunits mediate the effect of nicotine on reward processing and reinforcement learning and are involved in nicotine dependence.

**Table 1:** Subtypes of nAChRs in midbrain dopaminergic system

Region	Neurochemistry	nAChR subunits	nAChR subtypes	Chronic nicotine
VTA	DA neurons	All have α2, α4, α5, α6, β2, β3	α4β2: α4α5α6β2	No change
		Some have α3, α7	α7	No change
		Few have β4	α3β4	No change
	GABA neurons	α4, β2, β3, α7	α4β2	Upregulation
			α7	No change
SNc	DA neurons	All have α2, α4, α5, α6, β2, β3	α4β2	No change
		Some have α3, α7	α4α5α6β2	No change
		Few have β4	α7	No change
	GABA neurons	α4, β2, β3, and α7	α4β2	Upregulation
			α7	No change
NAcc	DA terminals	α4, α6, β2, β3	α4α6β2	Downregulation
			α6β2β3	Downregulation
CPu	DA terminals	α4, α5, α6, β2, β3	α4α6β2β3	Downregulation
			α4α6β2	Downregulation
			α4α5(non-α6)β2	Upregulation

Currently available drugs for smoking cessation include nicotine, partial agonists of nAChRs (cytisine and varenicline), and bupropion [106]. Partial agonists are used because they can moderately activate nAChRs to obtain normal levels of dopamine to reduce nicotine withdrawal symptoms, but the increase in dopamine levels is insufficient to cause satisfaction as strong as that induced by nicotine [107]. Among these drugs, varenicline, a partial agonist of β2-containing nAChRs and a full agonist of α7- and α3β4-containing nAChRs, has the highest success rate for smoking cessation [106]. On the one hand, acute varenicline can mildly activate α4β2- and α6β2-containing nAChRs, and α7 nAChRs, attenuating nicotine withdrawal symptoms and reducing motivation for nicotine intake [108]. On the other hand, chronic varenicline upregulates α4β2 nAChRs in a manner similar to that of chronic nicotine, but it also upregulates α3β4 and α7 nAChRs [109]. This pattern may mitigate dysfunction of the midbrain circuit following the selective upregulation of α4β2 nAChRs in GABAergic neurons by chronic nicotine.

Therefore, α4β2- and α6β2-containing nAChRs in the midbrain DA system are effective targets for smoking cessation. However, other subunits with relatively lower expression levels, such as the α7, α3, α5, β3, and β4 subunits, should be considered in future investigations because these subunits have been reported to regulate the α4, α6, and β2 subunits. Novel drugs that are more specific for nAChR subtypes should be designed to minimize the unpleasant side-effects of currently available drugs for smoking cessation, including nausea and depressed mood [1].
